# Cucumber Metallothionein-Like 2 (CsMTL2) Exhibits Metal-Binding Properties

**DOI:** 10.3390/genes7120106

**Published:** 2016-11-30

**Authors:** Yu Pan, Yanglu Pan, Junpeng Zhai, Yan Xiong, Jinhua Li, Xiaobing Du, Chenggang Su, Xingguo Zhang

**Affiliations:** 1 Key Laboratory of Horticulture Science for Southern Mountainous Regions, Ministry of Education, College of Horticulture and Landscape Architecture, Southwest University, No. 2 Tiansheng Road, Beibei, Chongqing 400715, China; pyl77111@163.com (Y.P.); pgu3456@163.com (J.Z.); ljh502@swu.edu.cn (J.L.); duxb163@163.com (X.D.); suchenggang@swu.edu.cn (C.S.); 2 Qijiang District Bureau of Forestry, Chongqing 401420, China; 3 Institute of Vegetables and Flowers, Chongqing Academy of Agricultural Sciences, Chongqing 401329, China; xiongyan7788@126.com

**Keywords:** cucumber, *CsMTL2*, metallothionein, metal-binding properties

## Abstract

We identified a novel member of the metallothionein (MT) family, *Cucumis sativus* metallothionein-like 2 (CsMTL2), by screening a young cucumber fruit complementary DNA (cDNA) library. The *CsMTL2* encodes a putative 77-amino acid Class II MT protein that contains two cysteine (Cys)-rich domains separated by a Cys-free spacer region. We found that *CsMTL2* expression was regulated by metal stress and was specifically induced by Cd^2+^ treatment. We investigated the metal-binding characteristics of CsMTL2 and its possible role in the homeostasis and/or detoxification of metals by heterologous overexpression in *Escherichia coli* cells. Furthermore, we produced a deletion mutant form of the protein, CsMTL2m, that contained the two Cys-rich clusters but lacked the spacer region, in *E. coli*. We compared the metal-binding properties of CsMTL2 with those of CsMTL2m, the β domain of human metallothionein-like protein 1 (HsMTXb), and phytochelatin-like (PCL) heterologously expressed in *E. coli* using metal-binding assays. We found that *E. coli* cells expressing *CsMTL2* accumulated the highest levels of Zn^2+^ and Cd^2+^ of the four transformed cell types, with levels being significantly higher than those of control cells containing empty vector. *E. coli* cells expressing CsMTL2 had a higher tolerance for cadmium than for zinc ions. These findings show that CsMTL2 improves metal tolerance when heterologously expressed in *E. coli*. Future studies should examine whether CsMTL2 improves metal tolerance in planta.

## 1. Introduction

Metallothioneins (MTs) are a family of low molecular weight polypeptides (7–10 kDa) with a high percentage (20%–30%) of cysteine (Cys) residues and metal-binding sites that occur in diverse organisms [1,2]. Moreover, MTs, one of the best-characterized classes of heavy metal-binding ligands in plant cells, maintain metal homeostasis and impart protection against heavy metal toxicity by intracellular sequestration [3,4,5]. MTs also play roles in other cellular processes of plants; for instance, they modulate cell growth and proliferation, DNA damage repair, the activity of metalloenzymes and transcription factors [6,7], the metabolism of metallodrugs, responses to stress conditions, and the scavenging of reactive oxygen species [7,8,9].

Based on their arrangement of Cys residues, MTs can be divided into three classes (I, II, and III) [1,7,10,11,12,13,14,15]. Class I MTs, which contain 20 highly conserved Cys residues, are widely distributed in vertebrates [16,17]; Class II MTs, which have a flexible arrangement of Cys residues, are encoded by a family of genes that are ubiquitous in plants, fungi, animals, and cyanobacteria [1,12,15,18]. Class III MTs consist of phytochelatins (PCs), another well-characterized family of heavy metal-binding ligands in plant cells. PCs are Cys-rich peptides with the general structure of (γ-Glu-Cys) *n*-Gly (*n* = 2–11), and are synthesized from glutathione (GSH) in a reaction catalyzed by PC synthase (PCS) [19,20]. Moreover, genes encoding MTs (referred to as MT-like genes) have been identified in plants [1], which have more diverse amino acid sequences and Cys residue arrangements than do mammalian MTs [21,22]. According to the position and allocation of Cys residues, MTs and MT-like proteins are further divided into four types in angiosperms [1,11,23,24,25]. Type 1 plant MT genes are more highly expressed in roots than in leaves; Type 2 plant MT genes are most strongly expressed in shoots; Type 3 plant MT genes are highly expressed in ripening fruits and leaves; and Type 4 plant MT genes are only expressed in developing seeds [1]. MTs and MT-like proteins have been identified in many plants, including *Oryza sativa* (rice) [26], *Arabidopsis thaliana* [27], *Citrullus lanatus* (watermelon) [28], *Solanum lycopersicum* (tomato) [29], and *Brassica juncea* [30]. Moreover, over-expressing or heterologously expressing plant MT genes could increase the tolerance of plants to bivalent metal ions such as zinc (Zn), copper (Cu), cadmium (Cd), and mercury (Hg). For example, *Arabidopsis* MT1 knock-down lines exhibit Cd sensitivity, and transgenic tobacco plants heterologously expressing *Arabidopsis* MT genes are resistant to Cd toxicity [12]. In addition, some plant MT2 genes have also been heterologously expressed in various organisms, and were found to impart increased tolerance to metals in *E. coli* [4,13,26,31].

In the present study, we isolated, cloned, and characterized a cucumber metallothionein gene, *Cucumis sativus* metallothionein-like 2 (*CsMTL2*). We investigated the metal-binding characteristics of CsMTL2 and its possible role in the homeostasis and/or detoxification of metals via heterologous expression in *E. coli* cells. To evaluate the metal-binding properties of CsMTL2, we compared metal accumulation in *E. coli* cells heterologously expressing a deletion mutant form of the protein, CsMTL2m, the β domain of human metallothionein-like protein 1 (HsMTXb), and phytochelatin-like (PCL). These findings suggest that *CsMTL2* is a candidate gene for improving metal tolerance in plants and for heavy metal phytoremediation.

## 2. Materials and Methods 

### 2.1. Gene Cloning and Plasmid Construction 

The complementary DNA (cDNA) of *CsMTL2* (GenBank accession number CK700734) was obtained from a young cucumber (*Cucumis sativus* L. Cs0301) fruit cDNA library according to the manufacturer’s instructions (CLONTECH Laboratories, Inc., Mountain View, CA, USA). A 238 bp fragment of *CsMTL2* containing the full coding sequence was amplified by Polymerase chain reaction (PCR) with PrimeSTAR HS DNA polymerase (TaKaRa Biotechnology, Dalian, China) using the primer pair *CsMTL2*-forward (5′-TCTTGCTGTGGCGGAAACTGTG-3′, based on the region immediately after the translation initiation codon ATG) and *CsMTL2*-reverse (5′-CTCGAGCTCATTTACAGGTGCATGG-3′). The PCR products were digested with the restriction enzyme *Sac* I, and the resulting 234 bp fragments were ligated into the vector pET32a (+), which was first digested with *Nco* I, filled in with the Klenow enzyme, and digested with *Sac* I. The blunted *Nco* I site provided an ATG codon for the recombined *CsMTL2*. The resulting plasmid, which was confirmed by PCR, enzyme digestion, and DNA sequencing (Sangon Co., Shanghai, China), was designated pET32a-*CsMTL2*.

*CsMTL2*m, an internal deletion variant of *CsMTL2*, was constructed to join the separated parts of the 5′ and 3′ ends encoding a high percentage of cysteine (Cys) residues together. The two parts were amplified from pET32a-*CsMTL2* using primers *CsMTL2*-F1 (5′-GTGAGCGGATAACAATTCCCCTCT-3′, located at the framework of pET32a (+)), *CsMTL2*-R1 (5′-TTCAAGCTTGCATCCTCCACAGCC-3′; the *Hin*d III site, located in the middle of *CsMTL2*, is underlined), *CsMTL2*-F2 (5′-TTGAAGCTTGGCTGCAAGTGCGGTGAC-3′; the *Hin*d III site, located in the middle of *CsMTL2*, is underlined), and *CsMTL2*-R2 [5′-CCAATCCGGATATAGTTCCTC-3′, based on a region within the framework of pET32a (+)], and the amplification products were digested with *Bgl* II, *Hin*d III, *Hin*d III, and *Sac* I, respectively. The resulting fragments (100 bp and 59 bp in length, respectively) were ligated into the larger fragment of pET32a (+) that had been digested with *Bgl* II and *Sac* I. The resulting plasmid was designated pET32a-*CsMTL2*m.

The human metallothionein-like protein 1 gene (*HsMTX*, GenBank accession number: CAA54136) was synthesized by the GeneRay Biotechnology Company (Shanghai, China). *HsMTXb*, encoding the β domain of HsMTX, was amplified using PrimeSTAR HS DNA polymerase (TaKaRa Biotechnology) with primers HsMTXb-forward (5′-CATGCCATGGACCCGAACTGCTCCTGCTCGCC-3′) and HsMTXb-reverse (5′-CTCGAGCTCAGCTGCAGCAGCTCTTCTTG-3′). The resulting 120 bp PCR product was digested with *Nco* I and *Sac* I, heated at 80 °C for 10 min to inactivate the enzymes and to melt the short truncated DNA, and immediately placed on ice to keep the truncated DNA in single-strand form. Then, the 112 bp targeted fragment was ligated into pET32a (+) that had been digested with *Nco* I and *Sac* I. The resulting plasmid was designated pET32a-HsMTXb.

Phytochelatin-like (PCL) was designed according to the structure of phytochelatin (γ-G1u-Cys)11-Gly. This protein was enzymatically synthesized in plants using an artificial gene encoding the deduced polypeptide MECECECECECECECECECECECG, which was abbreviated as Met-(α-G1u-Cys)11-Gly, harboring 11 (45.83%) Cys residues. *PCL* was also recombined into pET32a (+) at the *Nco* I and *Sac* I sites, resulting in the plasmid pET32a-*PCL*.

### 2.2. qRT-PCR Analysis of *CsMTL2* Expression under Zn^2+^ and Cd^2+^ Stress

Cucumber plants were grown in 9 cm pots containing a mixture of organic substrate and vermiculite (3:1) in a standard growth room. Six-week-old cucumber plants were placed into trays with different concentrations of ZnSO_4_ (0.05, 0.1, 0.5, 1.0, and 2.0 mM) or CdCl_2_ (0.05, 0.1, 0.2, and 0.3 mM). Leaves, stems, and roots were collected at 0 h, 6 h, 12 h, 24 h, 2 days, and 4 days. All plant tissues used for total RNA preparation were collected at the same time each day, frozen in liquid nitrogen, and stored at −70 °C until use.

Total RNA was extracted using an RNA prep Pure Plant Kit (TIANGEN, Beijing, China). The RNA was quantified using a NanoDrop (Gene Co., Beijing, China). First-strand cDNA synthesis was performed with 1 µg of total RNA using a PrimeScript RT Reagent Kit with a gDNA Eraser (TaKaRa Biotechnology, Dalian, China), according to the manufacturer’s instructions. RT-PCR was performed on a CFX96 Real-Time PCR system (Bio-Rad, Hercules, CA, USA) with Eva Green SMX (Bio-Rad), and the products were confirmed by 2.0% agarose gel electrophoresis. The primers were designed using Primer 5 software (Carnegie Institution of Washington, Stanford, USA). *Actin* was used as a control to normalize the quantitative reverse transcription (qRT)-PCR data across different samples (GenBank accession number: DQ115883) [32,33,34]. The primers used to amplify *Actin* were as follows:

CsActin-Q-F: 5′-GGTGGTGAACATGTAACCTC-3′,
CsActin-Q-R: 5′-TTCTGGTGATGGTGTGAGTC-3′,

The primers for *CsMTL2* were as follows:

CsMTL2-Q-F: AATGAGCAGCAGCAGCATCAAGAGC
CsMTL2-Q-R: AAGACGCAACACAACGGAGAACGAA

### 2.3. Expression and Purification of CsMTL2, CsMTL2m, HsMTXb, and PCL 

To express the recombinant proteins, the abovementioned plasmids were introduced into protease-defective and T7 RNA polymerase-containing *E. coli* strain BL21 (DE3). BL21 (DE3) cells transformed with pET32a (+) served as the control. *E. coli* BL21 (DE3) cells harboring MT genes in the pET32a (+) vector were grown at 37 °C overnight in the presence of 100 mg·L^−1^ ampicillin. The cultures were then transferred to fresh Lysogeny broth (LB) medium (1%, *v*/*v*) and grown to an Optical density (OD) 600 of 0.6 to 0.8. When this OD was attained, isopropyl-β-D-thiogalactopyranoside (IPTG) was added to the culture medium at a final concentration of 1 mM, and the culture was grown at 37 °C for 3 h. The cells were used for protein purification and immunoblot analysis (data not shown). The four metallothionein-like proteins were purified using the MagneHis^TM^ Protein Purification System (Promega, Shanghai, China). The four fusion proteins in the sample buffer (5% ME, 2% SDS, and 62.5 mM Tris-HCl, pH 6.8) were heated at 100 °C for 10 min and separated by 15% sodium dodecyl sulfate-polyacrylamide gel electrophoresis (SDS-PAGE).

### 2.4. Immunoblot Analysis

Protein separation by 15% SDS-PAGE and immunoblotting were performed as described previously. The proteins in the gel were transferred to a polyvinylidene difluoride (PVDF) membrane. The membrane was washed with a phosphate-buffered saline (PBS) buffer and incubated with a blocking buffer containing 5% (*v*/*v*) skim milk, washed again with blocking buffer, and incubated in a buffer containing anti-His, followed by incubation in a buffer containing a 1/5000 S-alkaline phosphatase-conjugated goat (Ig) G antibody as the secondary antibody.

### 2.5. Assay of Heavy Metal Binding Ability

To assess the heavy metal binding capacity of the four fusion proteins, *E. coli* cells transformed with pET32a-CsMTL2, pET32a-CsMTL2m, pET32a-HsMTXb, and pET32a-PCL were induced with isopropyl β-d-1-thiogalactopyranoside (IPTG) in 100 mL flasks as described above. Thirty minutes after IPTG induction, the flasks were supplemented with CdCl_2_ and ZnCl_2_ at a final concentration of 300 μM. Following induction, 0.05 g *E. coli* cell samples were placed into 50 mL porcelain crucibles and heated in a muffle furnace at 500 ± 25 °C. After 8 h, a small amount of mixed acid (HNO_3_:HClO_4_ = 3:4) was added to each crucible, which had been cooled to room temperature. The crucibles were heated again under gentle heat until no carbon residue was visible, followed by the addition of 10 mL of 8.3% hydrochloric acid to dissolve any remaining residue. The liquid was transferred to a 25 mL bottle; 8.3% hydrochloric acid was used as a blank control. This step was repeated three times. The amount of metal bound by the fusion proteins was analyzed by flame atomic absorption spectrometry.

### 2.6. Statistical Analysis

The heavy metal binding ability assay was performed in triplicate, and the standard error of the mean was calculated. Differences between the control and treatments were examined for statistical significance using a Student’s *t*-test implemented with SPSS15.0 software (SPSS Inc., Chicago, IL, USA).

### 2.7. Protein Analysis

Amino acid sequences were deduced and analyzed using ProtParam [35]. The amino acid sequences of the MTs were compared with sequences in the Protein Data Bank (PDB) database and Conserved Domain Data Bank (CDD) using BLASTP [36]. Amino acid sequences were aligned using ClustalX with default parameters [37].

## 3. Results

### 3.1. Isolation of *CsMTL2* cDNA and Structural Analysis of the Encoded Gene Product

**Figure 1:**
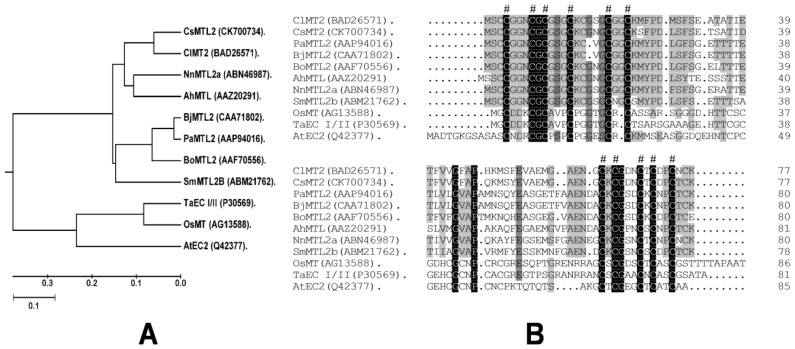
Phylogenetic tree of 11 selected plant metallothionein (MT) proteins and alignment of the deduced amino acid sequence of *Cucumis sativus* metallothionein-like 2 (CsMTL2) with MT proteins of other plants. (**A**) Phylogenic comparison of the deduced amino acid sequences of plant MT proteins. Scale bar denotes 0.1 amino acid substitutions per site. Accession numbers for the MT proteins used are included with the protein name. The MT genes are from *Arabidopsis thaliana* (At), *Triticum aestivum* (Ta), *Oryza sativa* (Os), *Brassica juncea* (Bj), *Brassica oleracea* (Bo), *Nelumbo nucifera* (Nn), watermelon (Cl), *Salix matsudana* (Sm), *Arachis hypogaea* (Ah), *Pringlea antiscorbutica* (Pa), and *Cucumis sativus* (Cs). (**B**) Comparison of the deduced amino acid sequence of CsMTL2 with the deduced amino acid sequences of plant MT proteins that have high levels of sequence similarity with CsMTL2. Amino acid residues that are conserved in at least eight of the eleven sequences are shaded, whereas those that are identical in all eleven proteins are in black. The hash symbol represents conserved Cys motifs.

We isolated *CsMTL2*, a 234 bp full-length cDNA sequence, from a cDNA library prepared from young cucumber fruits. The largest open reading frame (ORF) encodes a 77 amino acid protein with a predicted molecular mass of 7.765 kDa (pI 4.53). Multiple sequence alignment of a variety of plant MT proteins, including those from *Arabidopsis thaliana* (At), *Triticum aestivum* (Ta), *Oryza sativa* (Os), *Brassica juncea* (Bj), *Brassica oleracea* (Bo), *Nelumbo nucifera* (Nn), *Citrullus lanatus* (Cl), *Salix matsudana* (Sm), *Arachis hypogaea* (Ah), *Pringlea antiscorbutica* (Pa), and *Cucumis sativus* (Cs), showed that CsMTL2 clustered with ClMT2, BoMTL2, NnMTL2, SmMTL2B, BjMTL2, and AhMTL in a phylogenetic tree (Figure 1A). Alignment of the deduced amino acid sequences of these proteins revealed the presence of conserved motifs, including Cys–Cys, Cys-X-Cys, and Cys-X-Y-Cys, which are located in both the N- and C-terminal regions, where X and Y are different amino acids and any amino acid other than Cys (Figure 1B). One Cys–Cys, one Cys-X-Y-Cys, and two Cys-X-Cys motifs were present in the N-terminal regions, while two Cys-X-Cys and one Cys-X-Y-Cys motifs were found in the C-terminal regions. CsMTL2 shares high levels of amino acid sequence identity with other type-2 metallothionein (MT)-like proteins, with the highest level of identity (90%) being shared with ClMT2 from watermelon (Figure 1B).

### 3.2. Expression Profiles of *CsMTL2* under Zn^2+^ and Cd^2+^ Stress

**Figure 2:**
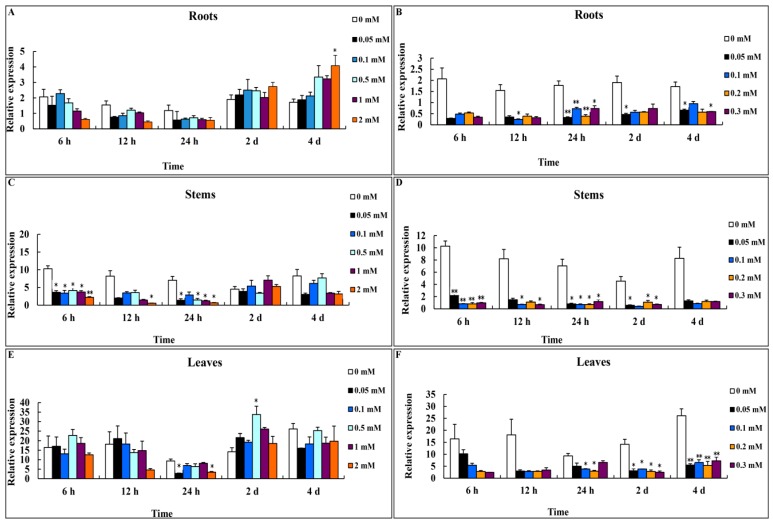
Expression profiles of *CsMTL2* in the roots, stems, and leaves of cucumber grown under Zn^2+^ and Cd^2+^ stress. *CsMTL2* expression in the roots, steams, and leaves of cucumber plants grown in the presence of different concentrations of Zn^2+^ (**A**,**C**,**E**) and Cd^2+^ (**B**,**D**,**F**) for the indicated periods, as detected by quantitative reverse transcription-polymerase chain reaction (qRT-PCR). Values represent the mean ± standard deviation (SD) of three biological replicates, with three technical replicates for each organ. Statistical significance was based on Student’s *t*-test ^*^: *p* < 0.05; ^**^: *p* < 0.01.

We analyzed the expression of *CsMTL2* after periods of exposure to various levels of Zn^2+^ and Cd^2+^ stress. For the Zn^2+^ treatments, six-week-old cucumber plants were grown in soil supplemented with 0 (H_2_O control), 0.05, 0.1, 0.5, 1.0, and 2.0 mM ZnSO_4_. Whereas the expression of *CsMTL2* was nearly unchanged in the control, except for a slight reduction at 24 h in the roots and leaves, and at 2 days in the stems, significant differences in expression were detected in stems during treatment (Student’s *t*-test, *p* < 0.01) (Figure 2A,C,E). In leaves, the expression of *CsMTL2* was upregulated at 2 days of Zn^2+^ treatment (Figure 2E). In roots, *CsMTL2* was downregulated at 6, 1, and 24 h and upregulated at 2 and 4 days of Zn^2+^ treatment (Figure 2A). The expression pattern of *CsMTL2* in stems was quite different from that in leaves and roots (Figure 2C). *CsMTL2* was downregulated at nearly all time points in response to different concentrations of Zn^2+^, especially at 6, 12, and 24 h of treatment (Figure 2C).

*CsMTL2* expression was downregulated when plants were grown in 0.05, 0.1, 0.2, and 0.3 mM CdCl_2_ (Figure 2B,D,F). There were no significant differences in the responses to different concentrations of CdCl_2_ in all organs tested. *CsMTL2* expression patterns in leaves, stems, and roots were similar. However, *CsMTL2* was more strongly expressed in leaves than in stems and roots (Figure 2F).

### 3.3. Protein Analysis

**Figure 3:**
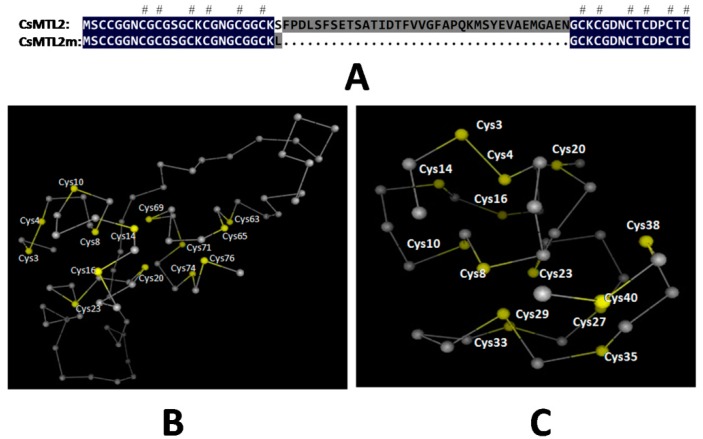
Schematic protein structure of CsMTL2 and CsMTL2m. (**A**) Comparison of deduced amino acid sequences of CsMTL2 and CsMTL2m. The blue background indicates shared amino acid residues. The gray background and dots denote differences between CsMTL2 and CsMTL2m. The hash symbol represents conserved Cys motifs. (**B**) Predicted 3D structure of-full length CsMTL2. Yellow and gray dots indicate the Cys motifs and other deduced amino acids co-linked by peptides (gray lines). (**C**) Predicted 3D structure of CsMTL2m, which lacks the linker region.

One of the most important characteristics of plant MTs is the presence of spacer regions, which are also referred to as linkers. These regions, which are devoid of Cys residues, connect individual domains with metal-thiolate clusters [38]. An analysis of the topology of SbMT-2 showed that this protein contains N-terminal and C-terminal motifs joined by a β-strand and an α-helix element [7]. The 3D structures of MTs are generally depicted with bound metal ions. It is possible to predict the 3D structures of MTs based on their sequences after resolving the correlation between cluster formation and folding in these proteins. We produced the CsMTL2m peptide, which is a truncated version of CsMTL2 consisting of 41 amino acids and a predicted molecular mass of 3.895 kDa (pI 7.92), and containing both the N- and C-terminal regions of CsMTL2, but lacking the linker domains located at amino acids 1–24, 29, and 61–77 of CsMTL2 (Figure 3A). We found that the 3D structures of CsMTL2 and CsMTL2m are quite different (Figure 3B,C), based on SWISS-MODEL analysis [39].

### 3.4. Expression and Purification of Trx-CsMTL2, Trx-CsMTL2m, Trx-HsMTXb, and Trx-PCL Fusion Proteins 

**Figure 4:**
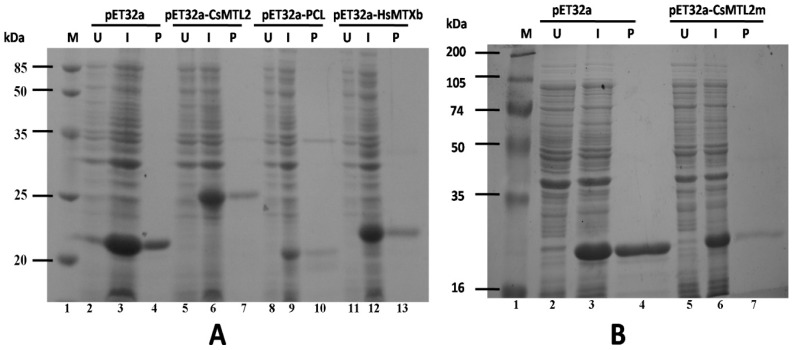
Sodium dodecyl sulfate-polyacrylamide gel electrophoresis (SDS-PAGE) analysis of Trx-CsMTL2, Trx-Pcl, Trx-HsMTXb, and Trx-CsMTL2m fusion protein heterologously expressed in *E. coli*. (**A**) Lane 1, molecular weight markers; lanes 2–13, proteins isolated from the transformed cells (pET32a (+), pET32a-CsMTL2, pET32a-PCL and pET32a-HsMTXb). U: before isopropyl-β-D-thiogalactopyranoside (IPTG) induction; I: IPTG induction; P: purified protein. (**B**) Lane 1, molecular weight markers; lanes 2–7, proteins isolated from transformed cells (pET32a (+) and pET32a-CsMTL2m).

Cells of *E. coli* strain BL21 (DE3) that were transformed with pET32a (+) as the control and induced with 1 mM IPTG produced a 20.397 kDa Trx-His protein (Figure 4A, lane 3) that was absent in non-induced cells (Figure 4A, lane 2). By contrast, cells transformed with pET32a-CsMTL2, pET32a-PCL, pET32a-HsMTXb, and pET32a-CsMTL2m produced Trx-CsMTL2, Trx-PCL, Trx-HsMTXb, and Trx-CsMTL2m fusion proteins, which were approximately 24.893 kD, 19.888 kD, 20.670 kD, and 21.019 kD in size, respectively, upon induction with IPTG (Figure 4A, lanes 6, 9, and 12; Figure 4B, lane 6), but not in the absence of IPTG (Figure 4A, lanes 5, 8, and 11; Figure 4B, lane 5). The purified Trx-His, Trx-CsMTL2, Trx-PCL, Trx-HsMTXb, and Trx-CsMTL2m proteins produced single bands on Tricine-SDS gels (Figure 4A, lanes 4, 7, 10, and 13; Figure 4B, lane 7). The expression of Trx-CsMTL2, Trx-PCL, Trx-HsMTXb, and Trx-CsMTL2m was further confirmed by immunoblot analysis using polyclonal antibodies, with untransformed bacterial protein and Trx serving as controls (data not shown).

### 3.5. Metal Tolerance and Ion Accumulation in E. coli Expressing CsMTL2, CsMTL2m, HsMTXb, and PCL

**Figure 5:**
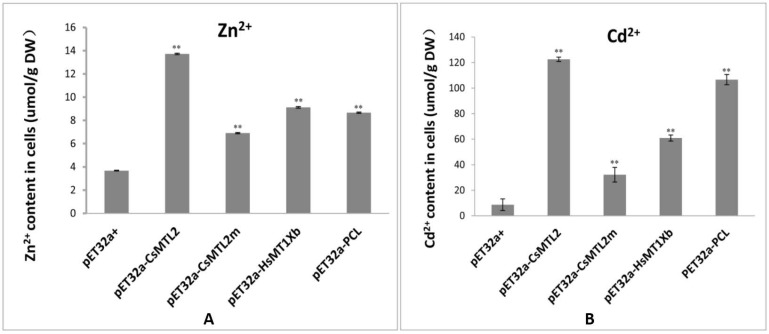
Metal ion accumulation in transgenic *E. coli*. Comparison of zinc (Zn) and cadmium (Cd) ion accumulation after 3 h of Zn^2+^ (**A**) and Cd^2+^ (**B**) ion treatment in *E. coli* cells heterologously expressing CsMTL2, CsMTL2m, HsMTXb, and PCL. The error bars represent standard errors of the mean from at least three independent experiments. Statistical significance was based on a Student’s *t*-test ^**^: *p* < 0.01. DW: dry weight.

We investigated the effects of Trx-CsMTL2, Trx-CsMTL2m, Trx-HsMTXb, and Trx-PCL expression on metal tolerance in *E. coli* in response to Zn^2+^ (0.3 mM ZnCl_2_) and Cd^2+^ (0.3 mM CdCl_2_), as MTs bind to a variety of metal ions, but preferentially bind to Zn^2+^ and Cd^2+^ ions [3,40,41]. Transformed cells containing pET32a-CsMTL2, pET32a-PCL, pET32a-HsMTXb, and pET32a-CsMTL2m were grown in LB medium supplemented with either Zn^2+^ or Cd^2+^ ions. Bacterial cells were harvested and the concentrations of accumulated metal ions (g^−1^ dry weight) were determined by flame atomic absorption spectrometry. *E. coli* cells expressing CsMTL2 exhibited the highest accumulation of Zn^2+^ ions among the four types of transformed cells, with levels being significantly higher than those of the control, pET32a (+) (Student’s *t*-test, *p* <0.01; Figure 5A). The levels of Zn^2+^ ions in *E. coli* cells expressing HsMTXb and PCL were similar, and those of cells expressing CsMTL2m were the lowest (Figure 5A). The levels of accumulated Cd^2+^ ions were significantly higher in *E. coli* cells expressing the four fusion proteins than in the control cells transformed with pET32a (+) (Student’s *t*-test, *p* < 0.01; Figure 5B). The highest levels of Cd^2+^ ions were detected in *E. coli* cells expressing CsMTL2, and the lowest levels were detected in cells expressing CsMTL2m. *E. coli* cells expressing PCL had higher Cd^2+^ levels than did cells expressing HsMTXb, with levels of PCL being closer to those of CsMTL2-expressing cells after 3 h of Cd^2+^ ion treatment. The ability of *E. coli* cells heterologously expressing the four fusion proteins to accumulate Cd^2+^ ions was significantly higher than their ability to accumulate Zn^2+^ ions (Student’s *t*-test, *p* <0.01; Figure 5A,B). *E. coli* cells expressing CsMTL2 accumulated Cd^2+^ ions at a rate that was 9.13-fold higher than that of Zn^2+^ ions. These results showed that *E. coli* cells expressing CsMTL2, CsMTL2m, HsMTXb, or PCL were significantly more tolerant to Zn^2+^ and Cd^2+^ than were cells transformed with pET32a (+). In addition, cells expressing CsMTL2, CsMTL2m, HsMTXb, or PCL were more tolerant to Cd^2+^ than to Zn^2+^ ions (Figure 5).

## 4. Discussion

Metallothioneins are Cys-rich, low molecular weight (4–8 kDa) metal-binding proteins that play important roles in many organisms. For instance, MTs maintain essential metal homeostasis and sequester toxic metals. Plant MTs commonly contain 14 conserved Cys residues and comprise ~80 amino acids [24,40]. In this study, we isolated *CsMTL2* from cucumber. We found that this gene encoded a putative protein with six Cys residues in the N-terminus and five in the C-terminus (Figure 1B). In addition, this gene encodes a deduced protein with the MSCCGGN sequence in its N-terminus (Figure 1B), which was also present in other plants, such as *Arabidopsis thaliana*, rice, and maize [42]. However, there are some variations among these MT2 protein sequences; for instance, AtMT2b contains MSCCGGS; PutMT2 from *P. tenuiflora* and AhMTL from *A. hypogaea* contain MSSCCGGN (Figure 1B); and SmMT2b from *S. miltiorrhiza* contains MSCCSGN [40,43]. Moreover, little is known about the properties of these variable sequences in MT2 proteins. It is possible that these variable sequences have specific functions, such as assisting with protein folding and stabilizing metal clusters [40,43]. Plant MTs play a crucial role in both metal metabolism and detoxification [20,44,45]. Moreover, studies of heavy metal accumulation by plants have focused on PC-mediated heavy metal uptake [46]. Further, the heterologous expression of the *Escherichia coli gshI* gene, which encodes γ-glutamylcysteine synthetase (γ-ECS), in Indian mustard (*Brassica juncea* L.) increased the biosynthesis of glutathione and PC, which enhanced Cd^2+^ tolerance and accumulation [30,47]. In rice, the GSH content and GST activity showed a significant correlation with Cd^2+^ levels [48]. In addition, *E. coli* cells overproducing fusion proteins of HbMT2 (*Hevea brasiliensis* metallothionein 2 protein) and QsMT2 (type II MT from *Quercus suber*) exhibit higher tolerances for copper and cadmium than do control cells [7,40,43]. In the current study, we found that the expression levels of *CsMTL2* were dramatically downregulated in cucumber roots, stems, and leaves when cucumber plants were exposed to various concentrations of Cd^2+^, but not when they were subjected to Zn^2+^ stress (Figure 2). In addition, we heterologously expressed some plant MT proteins in *E. coli* to compare their metal-binding properties. We found that the levels of Cd^2+^ ions were higher than those of Zn^2+^ ions in *E. coli* expressing pET32a (+), indicating that expression of *CsMTL2* could enhance a cell’s tolerance to Cd^2+^ ions and promote accumulation of Cd^2+^ (Figure 5). In accordance with this, the MTs have stronger properties of Cd detoxification [7,40,41,43]. Moreover, as Cd-binding proteins, MTs were first isolated from horse kidney, and subsequently identified in other animals, plants, fungi, and some bacteria [1]. In addition, MTs are thought to protect against Cd toxicity and maintain Zn homeostasis in mammals [10]. Therefore, further studies are required to confirm these results and to clarify the function of *CsMTL2* in plants.

In addition, plant MTs have a number of distinct functions that are influenced by the conserved arrangements of Cys residues and their tissue-specific expression [23,49]. The phylogenetic analysis conducted in our present study suggests that CsMTL2 belongs to the Type II MT plant protein family. Therefore, in an effort to characterize the metal-binding properties of Type 2 MTs, we compared a Type II plant MT (CsMTL2), a Type I MT (HsMTXb), and a Type III MT (PCL) in *E. coli* cells, and found that these proteins increase the Zn^2+^ and Cd^2+^ binding ability of *E. coli* (Figure 5). Moreover, *CsMTL2* might have a greater ability to accumulate Zn^2+^ and Cd^2+^ and a greater tolerance to these metals than *HsMTXb* and *PCL*, in accordance with the fact that MTs are directly involved in heavy metal detoxification and homeostasis [1]. Under the same conditions, however, the metal-binding ability of PCL (Type III MT) was also higher than that of HsMTXb (Type I) (Figure 5). Although these results seem to indicate that the arrangement and high content of Cys residues enable MTs to interact with metallic ions, snail MTs harboring the same number of Cys residues located in the same positions were shown to exhibit different metal binding behavior [50,51]. Therefore, further studies are needed to decipher the mechanism underlying metal-MT specificity in different species.

MTs are generally depicted as being bound to different metal ion species and the 3D structures of MTs can be predicted from their amino acid sequences after determining the folding patterns of MTs and the spatial relationship between clusters [38,52]. In the present study, topological analysis showed that N-terminal and C-terminal motifs of CsMTL2 were joined by a β strand and α helix element (Figure 3). Although evidence suggested that Cys residues were associated with the metallic ions of MT proteins, the concentrations of Zn^2+^ and Cd^2+^ were more significantly reduced in pET32a-CsMTL2m cells than in pET32a-CsMTL2 cells (Figure 5). Compared to the control, *E. coli* cells heterologously expressing CsMTL2m accumulated more Zn^2+^ or Cd^2+^ than did untransformed control cells, but much less Zn^2+^ or Cd^2+^ than cells expressing full-length CsMTL2 (Figure 5). These findings suggest that the linker region of CsMTL2 plays a crucial role in the metal-binding ability of MTs. Hence, further studies are needed to decipher the underlying molecular mechanisms that lead to the altered accumulation of metal between CsMTL2 and CsMTL2m. This work lays a foundation for defining the roles of *CsMTL2* in metal accumulation and detoxification.

## 5. Conclusions 

In this study, we revealed that *E. coli* cells expressing CsMTL2, CsMTL2m, HsMTXb, and PCL had higher tolerances to Zn^2+^ and Cd^2+^ than did control cells [pET32a (+)]. Heterologous expression of *CsMTL2* increased metal ion sequestration in *E. coli* cells, and the expression of a modified protein (CsMTL2m) lacking the linker region also increased the Zn^2+^ or Cd^2+^ binding ability of the transgenic *E. coli* cells. The linker region of CsMTL2 is important for both determining the protein structure and for sequestering metal ions.
