# Erratum: Pan Y. et al. Cucumber Metallothionein-Like 2 (CsMTL2) Exhibits Metal-Binding Properties. *Genes* 2016, *7*, 106

**DOI:** 10.3390/genes8010029

**Published:** 2017-01-12

**Authors:** Genes Editorial Office

**Affiliations:** MDPI, St. Alban-Anlage 66, 4052 Basel, Switzerland; genes@mdpi.com; Tel.: +41-61-683-77-34

The authors wish to make the following correction to their paper [1]. The funding number in the Acknowledgement Section is wrong. The correct number is Chongqing Graduate Scientific Innovation project (Project No.: CYB2015065). The authors would like to apologize for any inconvenience caused. The change does not affect the scientific results. The manuscript will be updated and the original will remain online on the article webpage.
